# Hormonal profile in early luteal phase after triggering ovulation with gonadotropin-releasing hormone agonist in high-responder patients

**DOI:** 10.3389/fendo.2022.834627

**Published:** 2022-08-15

**Authors:** Bella Martazanova, Nona Mishieva, Irina Vedikhina, Anastasia Kirillova, Irina Korneeva, Tatyana Ivanets, Aydar Abubakirov, Gennady T. Sukhikh

**Affiliations:** Federal State Budget Institution (FSBI) National Medical Research Center for Obstetrics, Gynecology and Perinatology named after Academician V.I. Kulakov Ministry of Healthcare of the Russian Federation, Moscow, Russia

**Keywords:** GnRH agonist trigger, dual trigger, modified luteal support, progesterone, OHSS

## Abstract

The major limitations associated with gonadotropin-releasing hormone agonist (GnRHa) triggering are inferior clinical outcomes in fresh embryo transfer cycles caused by luteal phase insufficiency following the GnRHa triggering. We included 153 high-risk patients in this study. In group I, the patients received gonadotropin-releasing hormone agonist (GnRHa) trigger + 1,500 IU human chorionic gonadotropin (hCG) support on the oocyte pick-up (OPU) day; in group II, the patients had a dual trigger (GnRHa + 1,500 IU hCG); and in group III (control), 10,000 IU hCG trigger was prescribed for the final oocyte maturation. The levels of LH, estradiol, and progesterone were evaluated in serum on the stimulation starting day, day 6 of stimulation, on the day of the trigger administration, OPU day, days 3 and 5 post-OPU, and day 14 post-ET, as well as in follicular fluid. Progesterone concentration was significantly lower in group I on OPU+5 compared to the hCG group (I vs. III, р = 0.0065). Progesterone levels were significantly lower in group II in serum on OPU+5 compared to groups I and III (I vs. II, *р* = 0.0068; II vs. III, *р* = 1.76 × 10^8^). The progesterone levels were significantly higher in follicular fluid in group III compared to the study groups (I vs. III, *р* = 0.002; II vs. III, *p* = 0.009). However, no significant differences in clinical outcomes were found between the groups. Then, we divided all women into pregnant and non-pregnant groups and found that estradiol (*p* = 0.00009) and progesterone (*p* = 0.000036*)* on the day of the pregnancy test were significantly higher in the pregnant women group. Also, progesterone on OPU day was significantly higher in the non-pregnant group (*p* = 0.033). Two cases of moderate ovarian hyperstimulation syndrome (OHSS) late-onset occurred in group I (3.5%, 2/56), no case of moderate/severe OHSS late-onset in group II, and three cases of moderate late-onset in group III (5.7%, 3/53). The low-dose hCG supplementation improves the luteal phase insufficiency after GnRHa triggering, which is confirmed by the comparable pregnancy rates in fresh transfer cycles between the groups. However, low-dose hCG carries a similar risk of OHSS as the full dose of hCG in high-responder patients.

## Introduction

The luteal phase insufficiency is leading to inferior clinical outcomes in fresh embryo transfer cycles after using gonadotropin-releasing hormone agonist (GnRHa) for oocyte maturation. This became the main limitation for GnRH triggering implementation in fresh cycles despite an effective avoidance of ovarian hyperstimulation syndrome (OHSS) (1). This negative outcome is the result of a luteal phase defect caused by the reduced luteinizing hormone (LH) surge and fast luteolysis (2). The GnRH agonist-induced surge lasts less than 48 h, which is crucial for corpus luteum support (3).

A recent Cochrane review demonstrated that GnRHa triggering is associated with low clinical pregnancy rates and high rates of early pregnancy loss (4, 5) despite good embryological outcomes (6–8). It is still an open question regarding the optimal protocol for luteal phase support after GnRHa triggering (9, 10). The main approaches that are used to overcome luteal phase insufficiency are i) luteal support with high doses of estradiol (E2) and progesterone (P) or ii) application of a single low-dose hCG bolus for the luteal phase rescue (10, 11).

According to current data, intensive luteal phase support is not enough for luteal phase insufficiency correction after GnRHa trigger in patients with peak E2 <4,000 pg/ml compared with those with peak E2 <4,000 pg/ml (11). The serum LH on the day of a trigger is one of the strictest predictors of pregnancy. The authors conclude that some form of LH supplementation after the GnRHa trigger may be necessary for corpus luteum support and *in-vitro* fertilization (IVF) success in high-risk patients with peak E2 <4,000 pg/ml (11). However, the LH/hCG supplementation in high-responder patients could increase the risk of OHSS development (12, 13).

This prospective observational study aimed to evaluate the levels of estradiol and progesterone and a low-dose bolus on OPU or dual trigger in high-risk patients after GnRHa agonist triggering versus the standard hCG trigger in fresh embryo transfer cycles.

## Materials and methods

### Study design

This study was a prospective observational study and was approved by the Ethics Committee and Institutional Review Board of Kulakov National Medical Research Centre of Obstetrics, Gynecology, and Perinatology, protocol no. 2, 15 February 2013. Written informed consent was provided by all participants. The patients were recruited from February 2013 to December 2014.

### Patient population

This is a secondary analysis of a study that was held in the years 2013–2014 in the Kulakov National Medical Research Centre of Obstetrics, Gynecology, and Perinatology. A total of 258 high-risk, high-responder patients were included. The inclusion criteria were as follows: age <40 years, anti-Müllerian hormone (AMH) level >2.5 ng/ml, antral follicle count (AFC) >14, and not using additional methods for OHSS prevention (such as dopamine agonist, GnRH antagonist in the luteal phase, etс.). The exclusion criteria were as follows: severe endometriosis, uterine abnormalities, subserosal fibroids, intramural fibroids >4 cm, hydrosalpinx, and severe male infertility. We excluded patients with an estradiol concentration >4,000 pg/ml (14,685.366 pmol/L) and patients that did not receive a fresh embryo transfer (ET). Thus, only 153 women were enrolled in the hormonal profile analysis (**Supplementary Materials**).

### Sample size

The power analysis was performed according to the data reported by Engmann et al. who demonstrated a 0% OHSS rate after GnRHa triggering and 31% in the hCG triggering control group (14). The 80% power of detection with a 31% difference between the group proportions was achieved with the group sample size of 20 with *L* = 0.025 (three-arm design).

Furthermore, a *post-hoc* power analysis was performed according to the results from Datta et al., who reported a 16.2% OHSS rate after GnRHa triggering and hCG bolus on OPU day and 31% in the hCG triggering group (12). The group sample size of 125 in the study groups with *L* = 0.025 (three-arm design) achieved 80% power of 31% difference between the group detection.

Power analysis was performed using the Statistica software first followed by using R. However, based on the totality of outcomes in the interim analysis, we decided to discontinue further recruitment.

### Outcomes

The primary outcomes were the serum and follicular concentrations of LH, estradiol, and progesterone and late-onset OHSS rate after different ways of ovulation triggering. The secondary outcomes were the number of mature metaphase II (MII) oocytes, the number of top-quality embryos per cycle, and the ongoing pregnancy rate.

### Treatment

The treatment description was accurately reported in our previous manuscript (13). All women underwent the IVF program using a flexible GnRH antagonist protocol. The patients were divided into three groups to receive one of the three types of ovulation trigger when at least three ovarian follicles had reached 17 mm in diameter.

In group I (*n* = 56), patients received the GnRHa trigger triptorelin 0.2 mg (Diferelin, Ipsen Pharma Biotech, Les Ulis, France) subcutaneously, and on the day of oocyte retrieval, 1,500 IU hCG (Pregnyl, Organon, Oss, the Netherlands) administered intramuscularly was added (15). In group II (*n* = 44), patients received a dual trigger (triptorelin 0.2 mg + 1,500 IU hCG) (16). In group III (*n* = 53), the control group, patients received a full-dose hCG trigger (10,000 IU Pregnyl, Organon, Oss, the Netherlands). The oocyte trigger was chosen by chance by a physician involved in the study.

All patients in groups I and II received luteal phase support with micronized P 600 mg/day (Utrogestan; Olvera, Spain; Besins Manufacturing, Belgium) and estradiol valerate 4 mg/day (Proginova; Lanno, France; Bayer Pharma AG, Germany) starting on the day after OPU. In group III, patients received micronized P 600 mg/day only, starting on the day after OPU.

In the study, we considered the late-onset forms of OHSS. Because all patients that were enrolled for analysis were allowed fresh ET and did not have early OHSS, the severity of OHSS was graded using the Golan classification (17). In the study, we considered moderate and severe forms of OHSS as they are clinically relevant.

### Embryology

The fertilization of mature oocytes (MII) was performed *via* standard IVF in case of normal sperm parameters according to World Health Organization recommendations (18) or by ICSI in case of male infertility. Embryos were cultured in 6% CO_2_ and 5% O_2_ in sequential media (ISM1, BlastAssist media; Origio, Denmark). Embryo transfer was performed either on day 3 (OPU+3) or day 5 (OPU+5). Blastocysts’ score was assigned according to the Gardner and Schoolcraft classification (19). Blastocysts graded ≥3BB on day 5 were classified as top-quality embryos. One or two embryos were used for the ET on OPU+3 or OPU+5.

### Hormonal level assessment

The hormonal level assessment was accurately reported in our previous manuscript (13). The serum concentrations of LH (IU/L), E2 (pmol/L), and P (nmol/L) were measured in real-time using an IMMULITE 2000 immunoassay system (Siemens AG, Flanders, NJ, USA). Plasma samples were obtained on the stimulation starting day, day 6 of stimulation, the day of ovulation triggering, OPU day, days 3 and 5 post-OPU, and the day of the pregnancy test.

Follicular fluid (FF) samples for hormonal level measurement were collected on the day of OPU. A total of 108 FF samples were obtained from 54 patients.

### Statistical analyses

Statistical analysis was performed with the IBM SPSS v 22.0. Continuous variables were tested for normality. For non-normally distributed data, we used the Kruskal–Wallis test and then the Mann–Whitney test. For the multiplicity of statistical testing, Bonferroni correction was applied. Categorical variables were compared using Fisher’s exact test. *p*-values <0.05 were considered statistically significant. Spearman’s rank correlation testing with *p*-values was defined as <0.05.

## Results

### Patients’ characteristics

Patients’ characteristics such as race, age, duration and causes of infertility, the starting and total doses of rFSH, mean basal E2 level, and mean AMH concentration did not differ significantly between the groups (**Table 1**).

**Table 1:** Demographic and stimulation data.

	GnRHa group (*n* = 56)	Dual trigger group (*n* = 44)	hCG group (*n* = 53)	*p*-value
Age, years	29 (27–33)	31 (28–33)	30 (29–33)	0.386
Body mass index, *n*	21.5 (20.3–24.55)	22.05 (19.6; 25.65)	22.2 (20.3–24.6)	0.874
Basal FSH, IU/L	6.15 (4.25; 7.33)	6.70 (5.3; 8.4)	6.2 (5; 8.1)	0.125
AMH, ng/ml	4.45 (3; 7.2)	3.60 (3.30; 5.06)	4.2 (2.85; 5.8)	0.351
AMH (2.5–4 ng/ml/>4 ng/ml), %	48.2%/51.8%	59.1%/40.9%	47.2%/52.8%	0.441
Infertility duration, years	4 (3–6)	3 (2; 4)	4 (3; 6)	0.095
Infertility I II	57.1 (32/56) 42.9 (24/56)	52.3 (23/44) 47.7 (21/44)	50.9 (27/53) 49.1 (26/53)	1.0
Infertility factors, % - Tubal -Male -PCOS -Combined -Unexplained	33.9 (19/56) 37.5 (21/56) 16.1 (9/56) 10.7 (6/56) 1.8 (1/56)	34.1 (15/44) 31.8 (14/44) 18.2 (8/44) 13.6 (6/44) 2.3 (1/44)	37.7 (20/53) 28.3 (15/53) 22.6 (12/53) 7.6 (4/53) 3.8 (2/53)	1.0 0.215
Starting rFSH dose, IU	150 (150; 200)	150 (150; 200)	150 (150; 200)	0.918
Total rFSH dose, IU	1,500 (1,200; 1,975)	1,350 (1,000; 1,762.50)	1,600 (1,200; 1,987.5)	0.294
Number of follicles ≥11 mm on ovulation triggering day, *n*	17 (15; 20)	19 (16; 20)	18 (16; 20)	0.064

Values are median (25%–75%) unless otherwise noted.

PCOS, polycystic ovarian syndrome.

### Serum hormone levels

The LH levels were significantly lower on the OPU day in the hCG group (I vs. III, *р* = 4.61 × 10^8^; II vs. III, *p* = 1.71 × 10^7^), and the same result was observed in follicular fluid, too (I vs. III, *р* = 0.002; II vs. III, *p* = 0.009). The estradiol concentration was similar in all groups during ovarian stimulation and was significantly lower in the dual trigger group on OPU+5 (I vs. II, *р* = 0.004; II vs. III, *p* = 0.001). Progesterone levels were significantly lower in the dual trigger group in serum on OPU+3 compared to the hCG group (II vs. III, *р* = 0.045); however, after the Bonferroni correction was applied, the significance was not confirmed (**Тable 2**).

**Table 2:** LH, estradiol, and progesterone concentrations in blood serum. .

Hormonal profile	GnRHa group (*n* = 56)	Dual trigger group (*n* = 44)	hCG group (*n* = 53)	*p*-value
Basal LH, (IU/L)	4.5 (3; 6)	5.1 (4; 6.9)	4.2 (3.2; 5.9)	0.289
LH on day 6 of stimulation, (IU/L)	4.6 (2; 11)	4.3 (2; 9)	3.7 (2; 7.4)	0.362
LH, triggering day (IU/L)	1.07 (0.5; 1.6)	0.70 (0.4; 1.7)	1.45 (0.6; 2.2)	0.160
LH, OPU	3.60 (1.8; 5.05)	3.40 (2.1; 4.9)	0.80 (0.55; 1.5)	**5.081 × 10^9^** ***p*_1/3_ = 4.61 × 10^8^** ***p*_2/3_ = 1.71 × 10^7^** ***p*_1/2_ = 1.0**
LH, OPU+3	0.30 (0.19; 0.5)	0.30 (0.20; 0.40)	0.40 (0.20; 1.0)	0.301
LH, OPU+5	0.2 (0.1; 0.5)	0.20 (0.1; 0.55)	0.1 (0.1; 0.6)	0.649
Basal estradiol, (pmol/L)	130.50 (90; 151)	105.00 (95; 163)	134.00 (104; 160)	0.470
Estradiol, on day 6 of stimulation, (pmol/L)	5,093.5 (3,043; 6,519)	4,582 (3,234; 5,724)	3,884 (3,256.5; 6,048.5)	0.916
Estradiol, triggering day (pmol/L)	9,034 (6,704; 11,266)	7,996 (6,138; 10,276)	8,704 (7,133; 10,442)	0.250
Estradiol, OPU	4,349 (2,928; 5,505)	3,172.00 (2,702; 4,148)	4,180 (2,750; 5,461)	0.066
Estradiol, OPU+3	6,321 (4,103; 9,130)	5,499.00 (3,783; 6,748)	5,136 (3,772; 5,878)	0.059
Estradiol, OPU+5	5,789.5 (4,119.5; 8,495.0)	3,190 (1,698; 5,668)	6,422.0 (5,180; 8,053)	**0.00011** ***p*_1/2_ = 0.004** ***p*_1/3_ = 1.0** ***p*_2/3_ = 0.001**
Estradiol, day of the pregnancy test	551 (346; 1,476)	683.00 (341; 1,208)	3,415 (406; 5,727)	0.234
Progesterone, triggering day (nmol/L)	2.85 (2; 3.4)	2.6 (1.7; 3.6)	2.55 (1.9; 3.35)	0.783
Progesterone, OPU	26 (17.1; 36)	28.30 (18.1; 39.8)	27.7 (19; 37.5)	0.853
Progesterone, OPU+3	321 (183.5; 371.5)	232 (162.5; 304)	304 (239; 368)	**0.045** ***p*_1/2_ = 0.205** ***p*_1/3_ = 1.0** ***p*_2/3_ = 0.052**
Progesterone, OPU+5	212.85 (124.5; 319)	97.0 (60.4; 178)	314.5 (268; 388)	**2.215 × 10^8^** ***p*_1/2_ = 0.0068** ***p*_1/3_ = 0.0065** ***p*_2/3_ = 1.76 × 10^8^**
Progesterone, day of pregnancy test	25.05 (4.8; 74.4)	58.5 (29.2; 77.6)	196.4 (28.3; 316)	0.220

Values are median (25%–75%) unless otherwise noted.

OPU, oocyte pick-up; LH, luteinizing hormone.

However, on OPU+5, progesterone concentrations were significantly lower in the dual trigger group compared to groups I and III (I vs. II, *р* = 0.0068; II vs. III, *р* = 1.76 × 10^8^) (**Table 2**). In group I, progesterone concentration was significantly lower compared to the control group on OPU+5 (I vs. III, *р* = 0.0065). The P levels were significantly higher in follicular fluid in the hCG group (I vs. III, *р* = 0.002; II vs. III, *p* = 0.009) (**Table 3**). Then, we divided all women into pregnant and non-pregnant groups and found that E (*p* = 0.00009) and P (*p* = 0.000036) on the day of the pregnancy test were significantly higher in the pregnant women group. Also, P on OPU day was significantly higher in the non-pregnant group (*p* = 0.033). The E2 on OPU+3 (*p* = 0.045) and OPU+5 (*p* = 0.047) was significantly higher in the pregnant women group; however, the *p*-value was near the 0.05 threshold (**Table 4**). The LH, estradiol, and progesterone concentrations in follicular fluid were comparable between the pregnant and non-pregnant women (**Table 5**). The LH, estradiol, and progesterone concentrations in blood serum and follicular fluid in non-pregnant and pregnant women per group are described in **Table 6**. The estradiol level was significantly higher in the blood serum of pregnant women compared to that of non-pregnant patients in all groups (group I = 0.042, group II = 0.028, group III = 0.022), and the progesterone concentrations were higher in the blood serum in the hCG group (*p* > 0.0001).

**Table 3:** LH, estradiol, and progesterone concentrations in follicular fluid.

	GnRHa group (*n* = 21)	Dual trigger group (*n* = 15)	hCG group (*n* = 18)	*p*-value
LH, IU/L	8.075 (3.8; 10.75)	11.60 (8.30; 16.8)	1.96 (1.6; 2.85)	**0.0000504** ***p*_1/2_ = 0.115** ***p*_1/3_ = 0.026** ***p*_2/3_ = 0.00002**
Estradiol, mmol/L	1.36 (0.705; 1.965)	0.87 (0.59; 1.51)	1.485 (1.0; 2.12)	0.278
Progesterone, mmol/L	17.05 (12.25; 23.61)	17.13 (13.78; 26.30)	35.04 (20.91; 60)	**0.0013** ***p*_1/2_ = 1.0** ***p*_1/3_ = 0.002** ***p*_2/3_ = 0.009**

Values are median (25%–75%) unless otherwise noted.

LH, luteinizing hormone.

**Table 4:** LH, estradiol, and progesterone concentrations in blood serum in pregnant and non-pregnant women.

Hormonal profile	Non-pregnant (*n* = 91)	Pregnant women (*n* = 62)	*p*-value
Basal LH, (IU/L)	5.1 (3.5; 6.3)	4.1 (3.2; 5.75)	0.125
LH on 6 days of stimulation, (IU/L)	4.20 (2; 10)	4 (1.90; 7.4)	
LH, triggering day (IU/L)	1.11 (0.55; 2)	0.9 (0.40; 1.80)	0.374
LH, OPU	2.55 (1.4; 4.6)	2.8 (1.1; 4.6)	0.961
LH, OPU+3	0.3 (0.13; 0.5)	0.3 (0.2; 0.5)	0.770
LH, OPU+5	0.2 (0.1; 0.55)	0.2 (0.1; 0.5)	0.823
Basal estradiol, (pmol/L)	120.0 (91; 147)	133.0 (104; 171)	0.121
Estradiol, on 6 days of stimulation, (pmol/L)	4,381 (3,130.5; 5,953.5)	4,582 (3,220; 6,391)	0.420
Estradiol, triggering day (pmol/L)	8,575 (6,685; 10,640)	8587 (6,633; 10,442)	0.942
Estradiol, OPU	3,838.0 (2,658; 4,915)	4,313.5 (3,067; 5,540)	0.067
Estradiol, OPU+3	5,222 (3,783; 6,538)	6,185 (4,258; 9,091)	**0.045**
Estradiol, OPU+5	5,092 (3,043.50; 6,083.50)	6,083.5 (4,020; 8,531)	**0.047**
Estradiol, day of the pregnancy test	406.0 (286; 773)	4,042.5 (1,113.5; 7,791)	**0.00009**
Progesterone, triggering day (nmol/L)	2.7 (1.80; 3.50)	2.6 (1.90; 3.40)	0.888
Progesterone, OPU	29.1 (21.9; 38)	22.8 (14.2; 33.7)	**0.033**
Progesterone, OPU+3	283.5 (182.25; 363.00)	303.5 (229.0; 368.5)	0.377
Progesterone, OPU+5	217.0 (76.3; 315.0)	228.5 (144.0; 324.0)	0.413
Progesterone, day of the pregnancy test	32.40 (12.3; 62.0)	246.0 (68.25; 423.95)	**0.000036**

Values are median (25%–75%) unless otherwise noted.

OPU, oocyte pick-up; LH, luteinizing hormone.

**Table 5:** LH, estradiol, and progesterone concentrations in follicular fluid in pregnant and non-pregnant women.

Hormonal profile	Non-pregnant (*n* = 38)	Pregnant women (*n* = 16)	*p*-value
LH, IU/L	7.98 (2.05; 11.6)	7.06 (2.225; 10.65)	0.734
Progesterone, mmol/L	20.11 (14.48; 33.96)	23.65 (14; 35.01)	0.844
Estradiol, mmol/L	1.3 (0.86; 1.86)	1.22 (0.74; 2.14)	0.793

Values are median (25%–75%) unless otherwise noted.

OPU, oocyte pick-up; LH, luteinizing hormone.

**Table 6:** LH, estradiol, and progesterone concentrations in blood serum and follicular fluid in non-pregnant and pregnant women per group.

	GnRHa group (*n* = 56)		*p*-value	Dual trigger group (*n* = 44)		*p*-value	hCG group (*n* = 53)		*p*-value
	Non-pregnant	Pregnant women		Non-pregnant	Pregnant women		Non-pregnant	Pregnant women	
LH, triggering day (IU/L)	1.07 (0.60; 1.50)	1.05 (0.50; 1.72)	0.943	0.90 (0.50; 1.70)	0.40 (0.50; 1.70)	0.254	1.65 (0.85; 2.35)	1.20 (0.50; 2.20)	0.350
LH, OPU	3.60 (1.60; 5.20)	3.83 (2.30; 4.80)	0.796	2.70 (1.90; 5.00)	4.00 (1.90; 5.00)	0.589	1.30 (0.60; 2.50)	0.60 (0.50; 0.80)	0.136
LH, OPU+3	0.30 (0.10; 0.50)	0.30 (0.20; 0.45)	0.568	0.30 (0.20; 0.40)	0.20 (0.20; 0.40)	0.627	0.30 (0.20; 1.40)	0.40 (0.20; 0.60)	0.739
LH, OPU+5	0.20 (0.10; 0.50)	0.20 (0.10; 0.60)	0.488	0.30 (0.10; 0.50)	0.20 (0.10; 0.50)	0.960	0.10 (0.10; 0.70)	0.10 (0.10; 0.50)	0.781
LH, follicular fluid, IU/L	7.90 (3.90; 11.90)	8.08 (3.70; 8.99)	0.602	11.60 (8.90; 14.90)	12.75 (8.90; 14.90)	0.851	1.96 (1.60; 2.98)	2.23 (1.80; 2.70)	0.750
Estradiol, triggering day (pmol/L)	9,104.00 (6,926.00; 11,230.00)	8,963.00 (6,182.00; 11,302.00)	0.980	8,517.50 (6,834.00; 10,675.00)	6.487.00 (6,834.0; 10.675.0)	0.195	8,622.50 (5,859.0; 10,640.0)	8,738.00 (7,344.0; 10,288.5)	0.509
Estradiol, OPU	4,332.50 (2,878.00; 5,488.00)	4,361.00 (3,109.00; 5,507.00)	0.711	3,081.00 (2,695.00; 4,042.50)	3.416.00 (2,695.0; 4,042.50)	0.150	4,082.00 (2,360.00; 5,445.00)	4,277.00 (3,067.00; 6,729.00)	0.287
Estradiol, OPU+3	6,009.50 (3,648.00; 7,583.00)	6,946.50 (5,719.50; 10,208.00)	0.069	5,401.00 (3,752.00; 6,662.50)	6,140.00 (3,752.00; 6,662.50)	0.371	5,135.50 (4,156.00; 5,555.00)	5,102.50 (3,440.00; 6,560.00)	0.779
Estradiol, OPU+5	5,257.00 (3,675.00; 7,305.00)	6,215.00 (5,103.00; 9,780.00)	0.099	3,086.50 (1,698.00; 5,668.00)	4,254.00 (1,698.00; 5,668.00)	0.327	6,410.50 (5,180.00; 8,117.00)	6,424.00 (4,508.00; 8,053.00)	0.912
Estradiol, follicular fluid, mmol/L	1.65 (0.71; 2.16)	0.94 (0.58; 1.42)	0.130	0.87 (0.72; 1.61)	1.27 (0.72; 1.61)	0.753	1.38 (1.11; 1.78)	2.14 (0.76; 2.36)	0.385
Estradiol, day of the pregnancy test	358.00 (230.00; 977.70)	9,817.00 (517.00; 17,239.00)	**0.042**	483.50 (313.50; 728.00)	1,286.00 313.50; 728.00)	**0**.**028**	403.00 (185.00; 910.00)	5,727.00 (3,415.00; 8,867.00)	**0**.**022**
Progesterone, triggering day (nmol/L)	2.60 (1.90; 3.30	3.00 (2.10; 3.50)	0.644	3.00 (2.15; 3.90)	2.50 (2.15; 3.90)	0.162	2.65 (1.50; 3.40)	2.50 (2.16; 3.30)	0.534
Progesterone, OPU	30.10 (22.00; 35.40)	20.50 (14.20; 26.60)	0.051	29.65 (20.05; 40.20)	22.45 (20.05; 40.20)	0.460	26.40 (21.90; 37.50)	32.40 (18.50; 34.70)	0.921
Progesterone, OPU+3	294.00 (124.35; 366.00)	326.00 (245.00; 380.50)	0.181	246.00 (171.00; 309.00)	231.00 (171.00; 309.00)	1.000	350.00 (240.00; 420.00)	287.35 (239.00; 334.00)	0.230
Progesterone, OPU+5	237.00 (125.00; 318.00)	180.00 (115.00; 353.00)	0.936	93.20 (57.40; 145.30)	146.20 (57.40; 145.30)	0.253	304.00 (268.00; 385.00)	324.00 (268.00; 407.00)	0.578
Progesterone, mmol/L	16.22 (11.37; 23.22)	18.97 (12.25; 23.65)	0.856	18.62 (15.42; 24.54)	14.19 (15.42; 24.54)	0.343	33.28 (18.67; 80.71)	35.04 (31.30; 36.79)	1.000
Progesterone, day of the pregnancy test	24.00 (4.50; 62.60)	307.50 (4.80; 478.90)	0.180	52.55 (25.65; 60.50)	73.65 (25.65; 60.50)	0.120	32.20 (13.15; 62.25)	316.00 (246.00; 485.00)	**>0**.**0001**

### Embryological and reproductive outcomes

There were no statistically significant differences in the main embryological (**Table 7**) or clinical outcomes between the groups (**Table 8**). No cases of fetal malformations were detected in this study. Three cases of premature birth on the 27–31 gestation weeks (one in group I and two in the hCG group) were observed. No significant difference was observed between the groups in moderate late-onset OHSS (**Table 8**).

**Table 7:** Embryological outcome.

	GnRHa group (*n* = 56)	Dual trigger group (*n* = 44)	hCG group (*n* = 53)	*p*-value
Oocytes, *n*	13 (11; 16)	12 (10; 14)	14 (12; 16)	0.147
MII oocytes, *n*	11 (9; 13)	10 (8; 12)	12 (10; 14)	0.242
2PN, *n*	8 (6; 11)	7 (5; 10)	10 (8; 11)	0.174
Fertilization rate (IVF)	77%	71%	79%	0.472
Fertilization rate (ICSI)	86%	78%	82%	0.374
Blastocysts, *n*	5.5 (3; 8)	4 (2.5; 6.5)	5 (2; 7)	0.153
Top-quality embryos, *n*	3 (1; 6)	3 (1; 4)	3 (0; 5)	0.349

Values are median (25%–75%) unless otherwise noted.

MII oocytes, metaphase II oocytes; 2PN, two-pronuclear zygote.

**Table 8:** Clinical outcomes and ovarian hyperstimulation syndrome rate.

	GnRHa group (*n* = 56)	Dual trigger group (*n* = 44)	hCG group (*n* = 53)	*p*-value
Positive hCG, % (*n*/per ET)	44.6% (25)	27.3% (12)	47.2% (25)	0.102
Clinical pregnancy, % (*n*/per ET)	39.3% (22)	27.3% (12)	41.5% (22)	0.305
Early pregnancy loss, % (*n*/per ET)	12.5% (7)	6.8% (3)	7.5% (4)	0.547
Delivery rate, % (*n*/per ET)	26.8% (15)	18.2% (8)	39.6% (21)	0.062
Moderate late-onset OHSS, %	3.5%, 2/56	0% (0/44)	5.7%, 3/53	0.292

ET, embryo transfer.

## Discussion

In this study, we compared the levels of estradiol, progesterone, and LH in the early luteal phase and the pregnancy test day after GnRHa triggering plus 1,500 IU hCG on OPU day (group I) and dual triggering (group II) approach with a full dose of hCG.

Our study indicates that estradiol and progesterone concentrations elicited by the modified luteal support including a small dose of hCG resulted in comparable pregnancy rates. However, low-dose hCG carries a similar risk of OHSS as the full dose of hCG in high-risk patients.

In the present study, we found a comparable late-onset OHSS in group I and the control group (with a full dose of hCG). Our previous results demonstrated that any dose of hCG caused a similar VEGF concentration in the blood, even when the GnRH agonist had been added for oocyte triggering, which could have resulted in OHSS (13) and supported the present conclusion.

According to previous data, the GnRHa triggering administration effectively eliminated OHSS in high-risk patients despite the hCG low-dose timing (20). Thus, no cases of OHSS were observed in the GnRHa group with the hCG support on OPU day in women at risk of OHSS with an E2 peak of 7,936 pmol/ml (20); only one case of late-onset OHSS was reported (*n* = 182) using the dual trigger approach despite the inclusion of high responders (a mean peak E2 level of 4,748 ± 1,493 pg/ml) (16). According to the latest publication, there was no difference in live birth rates and OHSS between patients who received low-dose hCG at the time of GnRH agonist trigger (dual trigger) and those who received low-dose hCG at the time of oocyte retrieval; however, the authors show that the cases of OHSS in the group who received low-dose hCG at the time of oocyte retrieval were moderate, while the one case of OHSS in the dual trigger group was mild (21).

However, some studies demonstrated a 16.2%–26% incidence of mild to moderate OHSS after GnRHa triggering following low-dose hCG luteal support on OPU day (12, 22) and a 9% incidence after the dual triggering in the high-responder patients (23).

After hormonal level analysis, we found that the LH concentrations were significantly lower on the OPU day in the hCG group (**Table 2**), and the same result was observed in follicular fluid, too (**Table 3**), which is the result of induced endogenous LH surge.

The progesterone levels were significantly lower on OPU+5 in the study groups compared to the hCG group (**Table 2**). We supposed that it is the result of corpus luteum luteolysis, even when low doses of hCG were added. However, the progesterone concentrations required for pregnancy in the fresh ET cycle were not known.

As the ovarian stimulation by itself and hCG as a trigger are not natural processes and induce superphysiological hormonal levels and multiple follicular growth with unnaturally long hCG corpus luteum support [the hCG surge lasts for 7–10 days after administration reaching a peak after 24 h (24) with a mean half-life of 2.32 days (55 h) (25)], the GnRH agonist approach is more natural for oocyte triggering, as it induces the FSH and LH surge which lasts only 24–36 h (24, 26).

The progesterone levels were significantly higher in follicular fluid in group III compared to the study groups (**Table 3**), which could be the result of the “truncated” endogenous LH surge and complement previous data on the importance of luteal phase modification after GnRHa triggering.

Then, we compared the LH, estradiol, and progesterone concentrations in blood serum and follicular fluid in non-pregnant and pregnant women per group (**Table 6**). No correlation was found between the studied parameters and the IVF outcomes during the early luteal phase. Only on the day of the pregnancy test, the estradiol level was significantly higher in the blood serum of pregnant women compared to that of non-pregnant patients in all groups (**Table 6**), while the progesterone concentrations were significantly higher in the blood serum in the hCG group than those in pregnant women (**Table 6**).

Thus, high-responder patients who had GnRH agonist triggering with further modified luteal support (groups I and II) had sufficient concentrations of estradiol and progesterone for pregnancy support, and even the levels of progesterone were significantly different between the groups on OPU+5 (**Table 2**).

Then, we divided all women into pregnant and non-pregnant groups, and as expected, we found that estradiol (*p* = 0.00009) and progesterone (*p* = 0.000036) on the day of the pregnancy test were significantly higher in the pregnant women group (**Table 4**). It is complementary to previously reported data (27) and could be the result of the endogenous hCG corpus luteum support.

Also, progesterone on OPU day was significantly higher in the non-pregnant group (*p* = 0.033). Despite that we did not find premature luteinization during stimulation in the present study, it is known that progesterone receptor expression in stimulated cycle endometria is similar to the one described during the first days of the luteal phase in natural cycles, and supraphysiological concentrations of steroid hormones might cause accentuated maturation of the endometrium in IVF cycles (28). So, we supposed that higher progesterone levels in the OPU phase could affect the endometrium.

The estradiol levels on OPU+3 (*p* = 0.045) and OPU+5 (*p* = 0.047) were significantly higher in the pregnant women group; however, the *p*-value was near the 0.05 threshold, so further studies are needed to evaluate this tendency (**Table 4**). The progesterone levels during this period did not show any difference between pregnant and non-pregnant women (**Table 4**).

All measured hormones in follicular fluid were comparable between pregnant and non-pregnant women (**Table 5**). We supposed that further investigations are needed to clarify the role of estradiol and progesterone in pregnancy prediction.

An interesting result of the influence of luteal serum progesterone levels on live birth rates was shown by Thomsen et al. (2018). The authors reported that serum progesterone levels of 60–100 nmol/L in the early luteal phase and 150–250 nmol/L during the mid-luteal phase correlated with the high chances of pregnancy in fresh embryo transfer cycles. Furthermore, mid-luteal progesterone levels >400 nmol/L led to a significant reduction in the chance of a positive hCG test, and patients with progesterone >100 nmol/L had a lower risk of an early pregnancy loss compared to the reference group (27). Also, according to a 2014 review, the minimum mid-luteal progesterone threshold is approximately 80–100 nmol/L, which correlates with an early pregnancy loss reduction and an increased live birth rate (29).

As a consequence, we ranged our results according to progesterone levels (1: progesterone levels of 60–100 nmol/L, 2: progesterone levels of 150–250 nmol/L, and 3: progesterone levels >400 nmol/L) on OPU+3 and OPU+5 and measured the correlation between pregnancy rates and early pregnancy loss; however, no difference was observed.

In line with the present study, the recent data from Kaye et al. demonstrated that corpus luteum function was higher when low-dose hCG was given on the OPU day compared with adjuvant hCG given on the ovulation trigger day. Though both methods of hCG support effectively improved the luteal phase insufficiency and led to high pregnancy rates, the authors concluded that the potential for OHSS risk with increased corpus luteum activity after hCG on the OPU day should be considered (30). Thus, they reported about three cases of mild–moderate OHSS in the group receiving adjuvant hCG on the OPU day (15%) and one in the group receiving the dual trigger (10%) (30). The authors supposed that the difference in hormonal profile between the groups with different timing of hCG supplementation could be the result of differences in corpus luteum age as the hCG-stimulated steroidogenic response is dependent on the age of the corpus luteum and steroidogenic acute regulatory gene expression (30, 31).

In the present study, we did not find any difference in the treatment success rates between the GnRHa (groups I and II) and the hCG triggering groups, and the reproductive outcomes of the study groups were similar to those of previous studies.

According to previous data, the ongoing pregnancy rate after GnRHa triggering plus the administration of a low dose of hCG on OPU day was reported to be 28%–37.1% (12, 20). Also, previous studies demonstrated the ongoing pregnancy rate of 57.7%–58.8% after dual triggering in fresh ET cycles (11, 16).

The current prospective randomized double-blind research that compared the timing of hCG (on ovulation triggering day or on the OPU day) supplementation after GnRHa triggering reported comparable live birth rates between the dual trigger group and the GnRHa group with hCG on OPU day (14/34, 41.2% vs. 21/37, 56.8%, *p* = 0.19), with OHSS rates of 9.7% and 3.8%, respectively (21).

The major limitations of this work are the small subset of patients and the non-randomization design. Also, another study limitation is that for the initial same size calculation, we used the early OHSS rate and not the serum hormonal concentrations and late-onset OHSS rate because late OHSS is not a common complication. In addition, in the study of Datta et al., no late-onset OHSS occurred in the GnRH agonist triggering and hCG groups; in contrast, the incidence of early mild-to-moderate OHSS was 16.2% with the GnRHa trigger and 31.0% with the HCG trigger (12). In the study of Humaidan et al., only two cases of moderate late-onset OHSS occurred in the hCG group, and there were no cases of late-onset OHSS in GnRH + 1,500 hCG in the high-risk patients (20).

However, our study is unique as it is one of the last studies that received approval from the ethics committee for hCG administration to high-risk patients. Moreover, the strength of the present study is that it involves good prognosis patients in terms of patient characteristics: young women with normal body mass index; high ovarian reserve without any burdened anamnesis, which reduces the effect of patient-dependent risk factors on the effectiveness of the IVF program; and the close monitoring of the patient’s hormonal response during ovarian stimulation and early luteal phase.

We believe that more studies are needed on luteal support avoiding low-dose hCG administration, with high doses of estradiol and progesterone only (10), because a single bolus hCG support for luteal phase rescue does not eliminate OHSS.

Based on the present data, we concluded that modified luteal support including a small dose of hCG effectively supports corpus luteum function, which results in similar pregnancy and OHSS rates compared to full-dose hCG in high-responder patients.

## Data availability statement

The raw data supporting the conclusions of this article will be made available by the authors, without undue reservation.

## Ethics statement

The studies involving human participants were reviewed and approved by Ethics committee and Institutional Review Board of Kulakov National Medical Research Centre of Obstetrics, Gynecology, and Perinatology protocol no. 2 from 15 of February 2013. The patients/participants provided their written informed consent to participate in this study.

## Author contributions

BM collected, analyzed, and interpreted the data and drafted the manuscript. NM was responsible for the conception, design, and data collection. IV was responsible for the collection and hormonal setting of the data. AK performed the embryological procedures and edited the manuscript. IK performed the OHSS treatment and reviewed and discussed the results. TI, AA, and GS reviewed and discussed the results. All authors contributed to the article and approved the submitted version.

## Conflict of interest

The authors declare that the research was conducted in the absence of any commercial or financial relationships that could be construed as a potential conflict of interest.

## Publisher’s note

All claims expressed in this article are solely those of the authors and do not necessarily represent those of their affiliated organizations, or those of the publisher, the editors and the reviewers. Any product that may be evaluated in this article, or claim that may be made by its manufacturer, is not guaranteed or endorsed by the publisher.

## References

[1] Fatemi HM Garcia-Velasco J. Avoiding ovarian hyperstimulation syndrome with the use of gonadotropin-releasing hormone agonist trigger. Fertil Steril (2015) 103:870–3. doi: 10.1016/j.fertnstert.2015.02.004 25724740

[2] Kol S. Luteolysis induced by a gonadotropin-releasing hormone agonist is the key to prevention of ovarian hyperstimulation syndrome. Fertil Steril (2004) 81:1–5. doi: 10.1016/j.fertnstert.2003.05.032 14711532

[3] Chandrasekher YA Hutchison JS Zelinski-Wooten MB Hess DL Wolf DP Stouffer RL. Initiation of periovulatory events in primate follicles using recombinant and native human luteinizing hormone to mimic the midcycle gonadotropin surge. J Clin Endocrinol Metab (1994) 79:298–306. doi: 10.1210/jcem.79.1.8027245 8027245

[4] Youssef MA van der Veen F Al-Inany HG Griesinger G Mochtar MH Aboulfoutouh I. “Gonadotropin-releasing hormone agonist versus HCG for oocyte triggering in antagonist assisted reproductive technology cycles,”. In: Youssef MA, editor. Cochrane database of systematic reviews (2011) 19;(1):CD008046. Chichester, UK: John Wiley & Sons, Ltd. doi: 10.1002/14651858.CD008046.pub3 21249699

[5] Youssef MAFM van der Veen F Al-Inany HG Mochtar MH Griesinger G Nagi Mohesen M. Gonadotropin-releasing hormone agonist versus HCG for oocyte triggering in antagonist-assisted reproductive technology. Cochrane Database Syst Rev (2014) 10:CD008046. doi: 10.1002/14651858.CD008046.pub4 PMC10767297 25358904

[6] Reddy J Turan V Bedoschi G Moy F Oktay K. Triggering final oocyte maturation with gonadotropin-releasing hormone agonist (GnRHa) versus human chorionic gonadotropin (hCG) in breast cancer patients undergoing fertility preservation: An extended experience. J Assist Reprod Genet (2014) 31:927–32. doi: 10.1007/s10815-014-0248-6 PMC4096878 24854484

[7] Bodri D Guillén JJ Galindo A Mataró D Pujol A Coll O. Triggering with human chorionic gonadotropin or a gonadotropin-releasing hormone agonist in gonadotropin-releasing hormone antagonist-treated oocyte donor cycles: findings of a large retrospective cohort study. Fertil Steril (2009) 91:365–71. doi: 10.1016/j.fertnstert.2007.11.049 18367175

[8] Papanikolaou EG Verpoest W Fatemi H Tarlatzis B Devroey P Tournaye H. A novel method of luteal supplementation with recombinant luteinizing hormone when a gonadotropin-releasing hormone agonist is used instead of human chorionic gonadotropin for ovulation triggering: A randomized prospective proof of concept study. Fertil Steril (2011) 95:1174–7. doi: 10.1016/j.fertnstert.2010.09.023 20979997

[9] Engmann L Benadiva C Humaidan P. GnRH agonist trigger for the induction of oocyte maturation in GnRH antagonist IVF cycles: A SWOT analysis. Reprod BioMed Online (2016) 32:274–85. doi: 10.1016/j.rbmo.2015.12.007 26803205

[10] Humaidan P Engmann L Benadiva C. Luteal phase supplementation after gonadotropin-releasing hormone agonist trigger in fresh embryo transfer: The American versus European approaches. Fertil Steril (2015) 103:879–85. doi: 10.1016/j.fertnstert.2015.01.034 25681859

[11] Griffin D Benadiva C Kummer N Budinetz T Nulsen J Engmann L. Dual trigger of oocyte maturation with gonadotropin-releasing hormone agonist and low-dose human chorionic gonadotropin to optimize live birth rates in high responders. Fertil Steril (2012) 97(6):1316–20. doi: 10.1016/j.fertnstert.2012.03.015 22480822

[12] Datta AK Eapen A Birch H Kurinchi-Selvan A Lockwood G. Retrospective comparison of GnRH agonist trigger with HCG trigger in GnRH antagonist cycles in anticipated high-responders. Reprod BioMed Online (2014) 29:552–8. doi: 10.1016/j.rbmo.2014.08.006 25246126

[13] Martazanova B Mishieva N Vtorushina V Vedikhina I Levkov L Korneeva I. Angiogenic cytokine and interleukin 8 levels in early luteal phase after triggering ovulation with gonadotropin-releasing hormone agonist in high-responder patients. Am J Reprod Immunol (2021) 85:1–9. doi: 10.1111/aji.13381 33247970

[14] Engmann L DiLuigi A Schmidt D Nulsen J Maier D Benadiva C. The use of gonadotropin-releasing hormone (GnRH) agonist to induce oocyte maturation after cotreatment with GnRH antagonist in high-risk patients undergoing *in vitro* fertilization prevents the risk of ovarian hyperstimulation syndrome: a prospective rando. Fertil Steril (2008) 89:84–91. doi: 10.1016/j.fertnstert.2007.02.002 17462639

[15] Humaidan P Ejdrup Bredkjær H Westergaard LG Yding Andersen C. 1,500 IU human chorionic gonadotropin administered at oocyte retrieval rescues the luteal phase when gonadotropin-releasing hormone agonist is used for ovulation induction: a prospective, randomized, controlled study. Fertil Steril (2010) 93:847–54. doi: 10.1016/j.fertnstert.2008.12.042 19200959

[16] Shapiro BS Daneshmand ST Garner FC Aguirre M Hudson C. Comparison of “triggers” using leuprolide acetate alone or in combination with low-dose human chorionic gonadotropin. Fertil Steril (2011) 95:2715–7. doi: 10.1016/j.fertnstert.2011.03.109 21550042

[17] Golan A Weissman A. Symposium: Update on prediction and management of OHSS - a modern classification of OHSS. Reprod Biomed Online (2009):28–32. doi: 10.1016/S1472-6483(10)60042-9 19573287

[18] World Health Organization. Laboratory manual for the examination and processing of human semen. Switzerland.:Cambridge Cambridge Univ Press (2010). Available at: https://apps.who.int/iris/handle/10665/44261

[19] Gardner DK Schoolcraft WB. Culture and transfer of human blastocysts. Curr Opin Obstet Gynecol (1999) 11:307–11. doi: 10.1097/00001703-199906000-00013 10369209

[20] Humaidan P Polyzos NP Alsbjerg B Erb K Mikkelsen AL Elbaek HO. GnRHa trigger and individualized luteal phase hCG support according to ovarian response to stimulation: Two prospective randomized controlled multi-centre studies in IVF patients. Hum Reprod (2013) 28:2511–21. doi: 10.1093/humrep/det249 23753114

[21] Engmann LL Maslow BS Kaye LA Griffin DW Diluigi AJ Schmidt DW. Low dose human chorionic gonadotropin administration at the time of gonadotropin releasing-hormone agonist trigger versus 35 h later in women at high risk of developing ovarian hyperstimulation syndrome - a prospective randomized double-blind clinical tri. J Ovarian Res (2019) 12:8. doi: 10.1186/s13048-019-0483-7 30684970 PMC6347742

[22] Seyhan A Ata B Polat M Son WY Yarali H Dahan MH. Severe early ovarian hyperstimulation syndrome following GnRH agonist trigger with the addition of 1500 IU hCG. Hum Reprod (2013) 28:2522–8. doi: 10.1093/humrep/det124 23633553

[23] O’Neill KE Senapati S Maina I Gracia C Dokras A. GnRH agonist with low-dose hCG (dual trigger) is associated with higher risk of severe ovarian hyperstimulation syndrome compared to GnRH agonist alone. J Assist Reprod Genet (2016) 33:1175–84. doi: 10.1007/s10815-016-0755-8 PMC5010813 27349252

[24] Fauser BC De Jong D Olivennes F Wramsby H Tay C Itskovitz-Eldor J. Endocrine profiles after triggering of final oocyte maturation with GnRH agonist after cotreatment with the GnRH antagonist ganirelix during ovarian hyperstimulation for *in vitro* fertilization. J Clin Endocrinol Metab (2002) 87:709–15. doi: 10.1210/jc.87.2.709 11836309

[25] Damewood MD Shen W Zacur HA Schlaff WD Rock JA Wallach EE. Disappearance of exogenously administered human chorionic gonadotropin. Fertil Steril (1989) 52:398–400. doi: 10.1016/S0015-0282(16)60906-8 2776893

[26] Itskovitz J Boldes R Levron J Erlik Y Kahana L Brandes JM. Induction of preovulatory luteinizing hormone surge and prevention of ovarian hyperstimulation syndrome by gonadotropin-releasing hormone agonist. Fertil Steril (1991) 56:213–20. doi: 10.1016/S0015-0282(16)54474-4 1906406

[27] Thomsen LH Kesmodel US Erb K Bungum L Pedersen D Hauge B. The impact of luteal serum progesterone levels on live birth rates-a prospective study of 602 IVF/ICSI cycles. Hum Reprod (2018) 33:1506–16. doi: 10.1093/humrep/dey226 29955789

[28] Papanikolaou EG Bourgain C Kolibianakis E Tournaye H Devroey P. Steroid receptor expression in late follicular phase endometrium in GnRH antagonist IVF cycles is already altered, indicating initiation of early luteal phase transformation in the absence of secretory changes. Hum Reprod (2005) 20:1541–7. doi: 10.1093/humrep/deh793 15705618

[29] Yding Andersen C Vilbour Andersen K. Improving the luteal phase after ovarian stimulation: Reviewing new options. Reprod BioMed Online (2014) 28:552–9. doi: 10.1016/j.rbmo.2014.01.012 24656557

[30] Kaye L Griffin D Thorne J Neuber E Nulsen J Benadiva C. Independent serum markers of corpora lutea function after gonadotropin-releasing hormone agonist trigger and adjuvant low dose human chorionic gonadotropin in *in vitro* fertilization. Fertil Steril (2019) 112:534–44. doi: 10.1016/j.fertnstert.2019.04.034 31227286

[31] Kohen P Castro O Palomino A Muñoz A Christenson LK Sierralta W. The steroidogenic response and corpus luteum expression of the steroidogenic acute regulatory protein after human chorionic gonadotropin administration at different times in the human luteal phase. J Clin Endocrinol Metab (2003) 88:3421–30. doi: 10.1210/jc.2002-021916 12843197

