# Supplementary material for: Hormonal profile in early luteal phase after triggering ovulation with gonadotropin-releasing hormone agonist in high-responder patients
Source: Front Endocrinol (Lausanne). 2022 Aug 15;13:834627. doi: 10.3389/fendo.2022.834627 (PMC9420862; doi:10.3389/fendo.2022.834627)

**SUPPLEMENTARY MATERIALS**

**Flowchart of the study design**

258 high-responder patients

Group III (n = 53)

a full dose hCG trigger

Group II (n=44)

a dual trigger group (GnRHa + hCG)

Group I (n = 56)

the GnRHa trigger+hCG on OPU

Patients with E2> 4000 pg/ml and/or no fresh ET

Group I (n = 91)

the GnRHa trigger+hCG on OPU

Group II (n=85)

a dual trigger group (GnRHa + hCG)

Group III (n = 82)

a full dose hCG trigger

**Graph of distribution of the number of follicles among the groups**


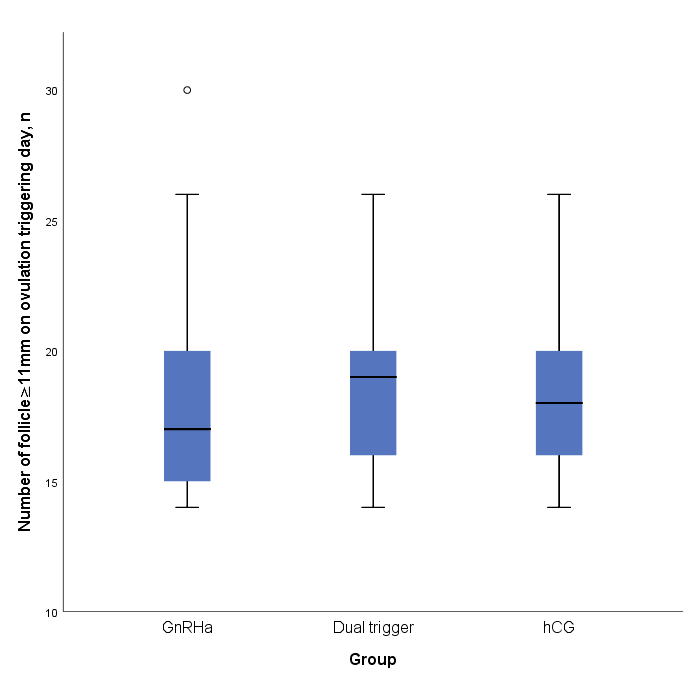

Supplement: Supplementary file 1 [file DataSheet_1.docx]
